# Effect of host microenvironment and bacterial lifestyles on antimicrobial sensitivity and implications for susceptibility testing

**DOI:** 10.1038/s44259-025-00113-3

**Published:** 2025-05-21

**Authors:** Ramon Garcia-Maset, Victoria Chu, Nicholas Yuen, Dalia Blumgart, Jenny Yoon, Benjamin O. Murray, Amelia A. Joseph, Jennifer L. Rohn

**Affiliations:** 1 Centre for Urological Biology, Department of Renal Medicine, Division of Medicine, University College London, London, WC1E 6BT UK; 2 Nottingham University Hospitals NHS Trust, Nottingham, NG5 1PB UK

**Keywords:** Microbiology, Infectious diseases, Bacterial infection

## Abstract

Bacterial infections remain a major global health issue, with antimicrobial resistance (AMR) worsening the crisis. However, treatment failure can occur even when bacteria show antibiotic susceptibility in diagnostic tests. We explore factors such as phenotypic resilience, bacterial lifestyles such as biofilms, and differences between laboratory tests and real infection sites, highlighting the need for improved platforms to better predict treatment outcomes, and reviewing emerging technologies aimed at improving susceptibility testing.

## Introduction and scope of review

Bacterial infections remain a significant source of morbidity and mortality despite advances in modern medicine. A recent report estimated their role in 7.7 million (13.6%) deaths worldwide regardless of antimicrobial resistance status—the second highest cause of mortality after ischaemic heart disease^1^. In parallel, antimicrobial resistance (AMR) is a growing crisis associated with 1.27 million deaths globally^2^. While AMR has received a significant amount of attention, treatment failure unrelated to bacterial AMR genes is far less understood.

The first step in microbiological diagnosis is identifying the causative organism(s), followed by determining susceptibility to antibiotics to guide prescribing. However, such in vitro susceptibility does not always translate to successful bacterial clearance or treatment outcome in the patient. We will discuss how treatment failure in infection with a susceptible microorganism is likely multifactorial, due to phenotypic resilience behaviours (e.g. tolerance and persistence) but also bacterial lifestyles such as biofilms; host factors; and environmental differences in diagnostic culture media compared with the in vivo infection site. Finally, we will examine emerging infection models that may better predict treatment outcomes, and offer future perspectives.

## The return of infection despite “appropriate” antibiotic treatment: terms of reference

**Fig. 1:**
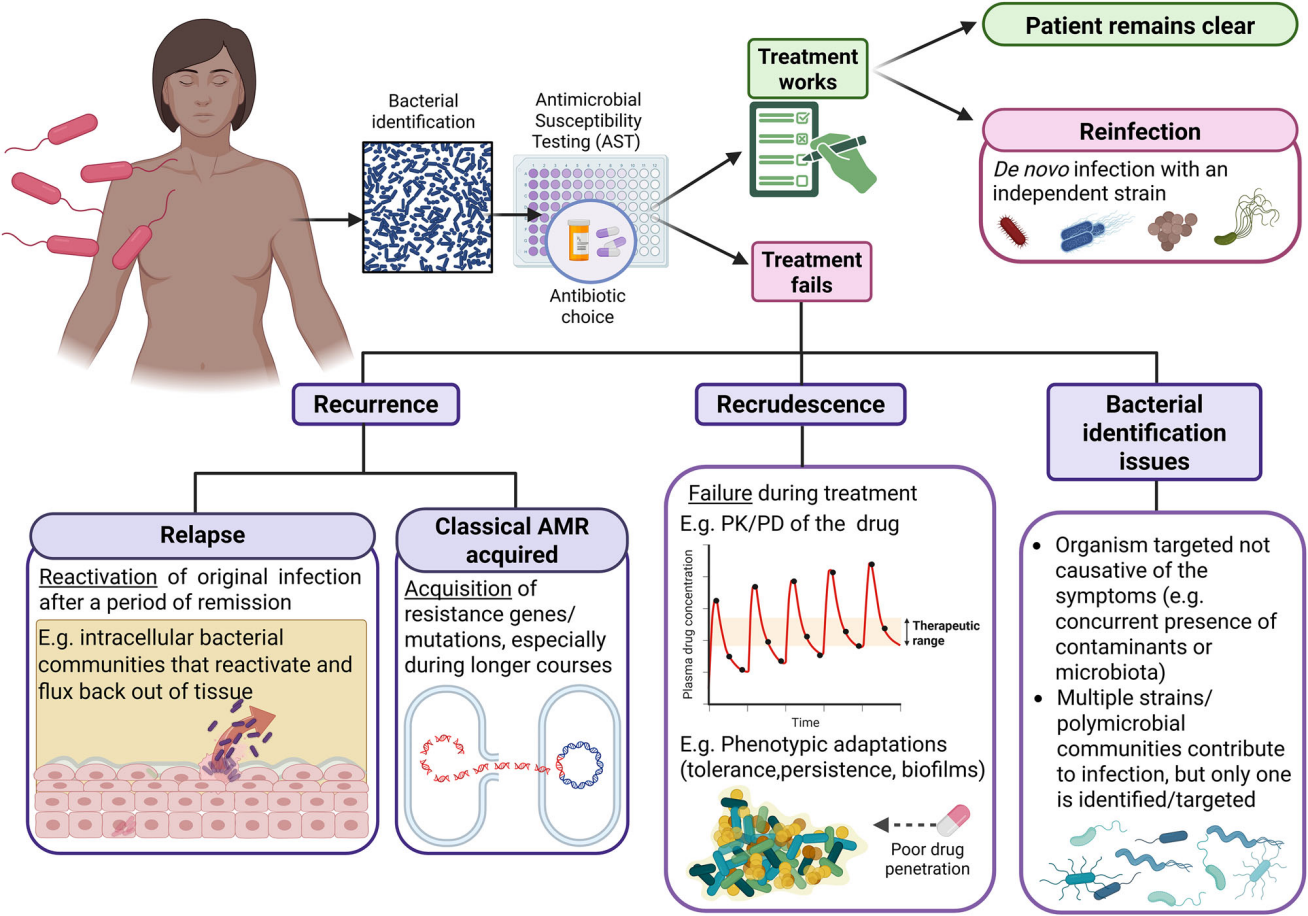
The wider context of treatment failure – terms of reference. Scheme presented is for an otherwise healthy individual (e.g. not immunosuppressed) with an acute infection.

Treatment failure despite antibiotic treatment represents a significant clinical challenge. As there are various related terms in the literature, we will first clarify the nomenclature we employ in this review (Fig. 1). We use the term **treatment failure** when referring to the inability of a drug to permanently clear the infection, no matter what the cause of failure. If measurable infection returns after treatment, this may be due to de novo infection of a new organism (**reinfection**), which is not a type of treatment failure. In the treatment failure category, one can describe **recrudescence** (outright failure of a drug to clear an infection *during* treatment). Alternatively, if the treatment initially succeeds but then the original agent returns, we deem this **recurrence**, which can be **relapse** (reactivation of the original infection from dormancy or sequestration in the body at some point *after* treatment) or the acquisition of classical antimicrobial **resistance**^3^, herein defined as an acquired bacterial genetic trait (e.g. an AMR gene such as beta-lactamase). This review will *not* deal with reinfection with a new strain, antibiotic resistance nor **intrinsic resistance** (e.g. membrane impermeability) that will never allow a drug to be efficacious in that particular bacterial class, e.g. cephalosporins against Enterococci^4^. We will also not discuss scenarios where the organism identified in the diagnostic laboratory (e.g. within a mixed growth) was not the organism causing the symptoms, and therefore treatment failed because the wrong bacteria was targeted by antibiotics. Instead, we will be discussing short- or long-term treatment failure in infections caused by bacteria that were predicted to be susceptible to a particular antimicrobial.

It is challenging to distinguish between true recurrence involving the same bacterial strain, and reinfection with a new strain of the same species with the same antibiogram (defined as the profile of susceptibility of a strain to an array of different drugs). To definitively determine the cause of a persisting infection, longitudinal sampling coupled with genotyping (preferably whole-genome sequencing) would be required, which is logistically challenging, resource-intensive in clinical settings, and rarely performed^5–8^. Hence, as a caveat, sometimes relapse and recrudescence are attributed as such without definitive proof. Therefore, this limitation should be kept in mind when we discuss the evidence below.

Another important point is that distinct terms are used within disease specialities which might differ. For instance, in the *Helicobacter pylori* infection community, recrudescence is used interchangeably with relapse, while re-infection is defined as infection with a new *H. pylori* strain after successful eradication. If infection returns after initially successful clearance within one year, it is known as recrudescence, while return after a year is called reinfection^9^. Regardless of terminology, treatment-resistant infections are associated with increased morbidity and mortality and can have significant quality of life impacts^10^. Often necessitating repeated or long-term antibiotic usage, they may also contribute to the growing issue of classical AMR^11^, which is predicted to cause ~10 million deaths attributable to or associated with AMR globally in 2050^2^.

## Mechanisms of treatment resistance

Traditionally, antimicrobial resistance is classified as intrinsic or acquired and is measured using the minimum inhibitory concentration (MIC) assay. MIC is currently the gold-standard methodology to guide clinical treatment decisions (Table 1). Using dilutions into liquid or agar, or gradient strips, visible inhibition of bacterial growth can be observed. The resulting clinical breakpoints indicate whether isolates are susceptible or resistant against a panel of antibiotics, often with designations for more intermediate or dose-dependent phenotypes depending on the framework^12,13^.

**Table 1:** Common antimicrobial sensitivity testing methods

Test	Definition	Advantages	Disadvantages
**Phenotypic Antimicrobial Sensitivity Testing (AST)**			
**Minimum Inhibitory Concentration (MIC)**	• Lowest concentration of an antibiotic that causes visible inhibition of bacterial growth^12^ • The broth microdilution method is the gold standard in the clinics^116,117^	• Easy, low cost and high-throughput methodology^118,119^ • Easy to standardise^118,119^ • Informative for clinical decision-making^118,119^	• Fails to discriminate between bacteriostatic and bactericidal^120,121^ • Static assay: cannot replicate complex dynamic interactions of human infections^122^ • Does not recapitulate infection microenvironment (host-pathogen interactions)^123^ • Requires previous bacterial isolation step, and bacterial growth required to read out (24h-48h)^124^ • Cannot distinguish if any resistance stems from planktonic or biofilm growth^16,95^
**Minimum Bactericidal Concentration (MBC)**	• Lowest concentration of antibiotic that causes a killing >99.9% of the bacterial population^125^ • Typically determined by colony forming unit (CFU) enumeration	• Low cost methodology^118,119^ • Easy to standardise^16^ • Discriminates between bacteriostatic and bactericidal^120^ • MBC/MIC ratios historically used to measure tolerance^120^	• Static system: cannot replicate complex dynamic interactions of human infections^124^ • Does not recapitulate infection microenvironment (host-pathogen interactions)^123^ • Requires previous bacterial isolation step and bacterial growth. and an additional step for CFU determination (2-3 days)^124^ • Cannot distinguish if any resistance stems from planktonic or biofilm growth^16,95^
**Time-killing assay**	• Bacterial population dynamics over time in the presence of antibiotic concentrations^126^	• Provides dynamics of microbial killing over time^121^ • Discriminates between bacteriostatic and bactericidal effects^121^ • Can be used to determine tolerance^14^ • Killing mechanism can be classified as time- or concentration-dependent^126^	• Requires multiple time points, each involving sampling, dilution and plating^127^ • Does not recapitulate infection microenvironment (host-pathogen interactions)^123^ • Does not directly identify mechanisms of resistance nor how bacteria may evolve to resist the antimicrobial agent • Only planktonic bacteria are tested, which may overestimate efficacy of antimicrobial agent compared with biofilms^16,95^
**Pharmaco-kinetics/ Pharmaco-dynamics (PK/PD)**	• Dynamic method allowing the addition of oxygen/ nutrient or drugs in a specific rate (dosing) to mimic an in vivo scenario^128^ • Typically involves a system of containers, tubing and a pump e.g. the hollow fibre bioreactor^129^	• Provides dynamics of microbial killing over time^130^ • Controls critical parameters (multiple drug doses over time)^130^ • Mimic some conditions found in human body, so a more accurate representation of antimicrobial effect^130,131^	• Requires multiple time points, each involving sampling, dilution and plating^130^ • While US FDA has approved hollow fibre bioreactor for in vivo-like studies, it does not fully capture the complexities of the human body^130^
**Omics based-AST methods**			
**Nucleic acid amplification tests (NAATs)**	• Allow the identification of bacterial species and known antibiotic resistance genes • PCR and quantitative PCR (qPCR) are the most common methods used^132^	• Rapidness and automation^124,133^ • Leads to an earlier diagnosis	• DNA isolation and purification required • Only known resistance mechanism can be detected^124^ • Elevated cost of specialist equipment • The identified AMR genes may not be directly associated with the pathogen responsible for the disease, or the detected resistance gene might not be functional^124^
**Whole genome sequencing (WGS)**	• Identifies pathogens (strain level) and predicts antimicrobial susceptibility from bacterial DNA sequences^119^	• Samples can be obtained directly from bodily fluids/ tissues (no bacterial culture required, significantly reducing readout time)^134^ • Allows study of bacterial evolution and data surveillance^135^ • Provides extensive characterization of resistance markers, plasmid replicons and virulence factors^135^	• Elevated cost of specialist equipment^124,135^ • Complex-associated bioinformatics increases turnaround time^124^ • No guarantee that flagged resistance genes expressed in all situations, nor that bacteria was alive^136^ • Resistance gene must be known to detect it^137^. • 2015 European Committee on Antimicrobial Susceptibility Testing (EUCAST) subcommittee concluded that evidence for WGS in establishing antimicrobial susceptibility was poor; absence of standardized protocols and guidelines confounds accurate/ consistent interpretation of results in the clinic^124,138^
**Mass spectrometry time-of-flight (MALDI-TOF)**	• Analyses molecular composition of proteins/ peptides by measuring their mass-to-charge ratio, can identify certain biomarkers at molecular level. When combined with a reference database, can be used to identify a pathogen and its resistance profile^139,140^	• Has transformed microorganism identification in routine clinical microbiology^141^	• Elevated cost of specialist equipment^139,140^ • Requires prior growth of a colony (additional time required)^139,140^ • Method usually coupled with an MIC assay, so certain limitations inherent in MICs are extrapolated to this methodology^142^

Less discussed is the subset of refractory phenotypes that may be masked by the MIC assay^14^. This section will describe these phenotypes and address the mechanisms responsible where known (summarised in Table 2 with additional examples). It should be noted that while the literature describes a vast array of mechanisms, here we focus on a selection deem to be particularly noteworthy within the context of this review. We also stress that there are multiple mechanisms (not all described in this review) that could result in a particular phenotype.

**Table 2:** Bacterial mechanisms of persistence through environmental factors, signalling and stress response and phenotypes (select examples, not meant to be exhaustive; additional examples are presented in the main text)

Component	Pathogen	Description
**Environmental Factors**		
**pH**	*Escherichia coli, Klebsiella* spp.	Lowering pH of bacterial growth medium enhances fosfomycin activity in majority of isolates studied^143^
	*Helicobacter pylori*	Lowering pH reduces activity of trospectomycin, ampicillin, metronidazole, clarithromycin, azithromycin and clindamycin, with macrolides and clindamycin most affected^144^
	*Escherichia coli*	Increasing pH improves erythromycin, gentamicin and ciprofloxacin efficacy, while lowering pH improves ampicillin efficacy^77^
**Salt and Urea**	*Escherichia coli*	Effects of salt and urea on antimicrobial efficacy are strain- and drug-specific. In some strain/drug combinations, higher salt and urea concentrations increase sensitivity; in others, they decrease it^145^
		Urea induces expression of genes coding for some *E. coli* virulence factors, such as capsule and fimbriae formation^146^
**Mucous**	*Pseudomonas aeruginosa*	Strains grown on a surface coated in mucin develop thicker biofilms that are more tolerant to tobramycin than those grown on glass, actin or DNA^147^
	*Pseudomonas aeruginosa and Staphylococcus aureus*	Glucose supplementation to artificial sputum promotes the growth of *P. aeruginosa* and *S. aureus* but has no significant effect in standard laboratory media^148^
**Antibiotic Exposure**	*Escherichia coli*	Pre-treatment with low doses of ciprofloxacin induces the SOS response and increases persister cell formation, leading to enhanced survival upon subsequent high-dose exposure^149^
	*Staphylococcus aureus*	Exposure to high concentrations of oxacillin, clarithromycin and moxifloxacin selects for a non-dividing persister state, which reverts back to a normal growth and antibiotic susceptibility after drug removal^150^
	*Enterococcus faecium, Staphylococcus aureus, Klebsiella pneumoniae, Acinetobacter baumannii, Pseudomonas aeruginosa, Enterobacter* spp. (ESKAPE pathogens)	Extended interval aminoglycoside treatment drives an increase in persister formation, leading to cross-tolerance to multiple antibiotics and persistence within biofilms without the emergence of genetic resistance^151^
**Signalling and Stress Response**		
**Two Component Systems**	*Pseudomonas aeruginosa*	Knocking out the GacS gene, a sensor kinase in the GacS/GacA two-component system, increases sensitivity to gentamicin, amikacin and chloramphenicol^152^
	*Staphylococcus aureus*	Inhibition of the GraRS two-component system reduces tolerance to daptomycin^70^
	*Enterobacter cloacae*	Meropenem treatment induces the PhoPQ two-component system, leading to outer membrane modifications that enhance bacterial survival and increase antibiotic tolerance^153^
**Quorum Sensing**	*Pseudomonas aeruginosa*	Azithromycin inhibits quorum sensing systems, reducing virulence factors, impairing oxidative stress resistance and limiting biofilm formation through decreased motility^154^
		By inhibiting quorum sensing with furanone, virulence factor production decreases, biofilm integrity is disrupted and biofilms become more susceptible to tobramycin and sodium dodecyl sulfate (SDS)^155^
	*Streptococcus mutans*	The stress inducible quorum sensing peptide CSP enhances the formation of multidrug-tolerant persister cells^156^
**Toxin/Anti-toxin Systems**	*Pseudomonas aeruginosa*	The ParE toxin inhibits DNA gyrase and in low concentrations, provides modest protective effects against antibiotics such as ciprofloxacin, levofloxacin and novobiocin^157^
**SOS Response**	*Escherichia coli*	Disrupting SOS stress response functions reduces persister levels by up to 43-fold following ciprofloxacin treatment compared with wild type strains^149^
	*Mycobacterium tuberculosis*	Depleting DNA gyrase, which in turn induces the RecA/LexA-mediates SOS response, leads to increased persister cell mediated tolerance to rifampicin, isoniazid and ethambutol^158^
**Stringent Response**	*Staphylococcus aureus*	Mutations in *S. aureus* that partially activate the stringent response cause growth defects that cause multidrug tolerance to ß-lactams, fluoroquinolones, glycopeptides and daptomycin^159^
	*Mycobacterium tuberculosis*	Deletion of *rel*_MTB_, the stringent response enzyme, prevents transformation into a quiescent state during nutrient starvation, leading to increased susceptibility to isoniazid^160^
**Phenotypes**		
**Biofilms**	*Klebsiella pneumoniae*	Cells enter a stationary phase induced by nutrient depletion within biofilms, which contributes to increased tolerance to ampicillin and ciprofloxacin^161^
	*Pseudomonas aeruginosa*	Longer treatment times and higher doses of colistin and imipenem are required to eradicate populations within biofilms compared with their planktonic states^36^
**Efflux Pumps**	*Mycobacterium tuberculosis*	Intracellular *M. tuberculosis* develops antibiotic tolerance within macrophages via the induction of bacterial efflux pumps, which is reversed by efflux pump inhibitors like verapamil^162^
	*Klebsiella pneumoniae*	The KpnEF efflux pump contributes to antibiotic tolerance, biofilm formation and stress survival^163^
**Intracellular Bacterial Communities**	*Salmonella enterica* serovar Typhimurium	*S. Typhimurium* forms intracellular bacterial communities within dendritic cells in the caecum-draining lymph nodes of mouse models, where they adopt a slow-growing, antibiotic tolerant state that allows survival against ciprofloxacin treatment^164^
**Membrane Vesicles**	*Pseudomonas aeruginosa*	The antibiotic-induced SOS response increases outer membrane vesiculation, enhancing bacterial survival under stress^165^
		Outer membrane vesicles facilitate biofilm formation by mediating quorum sensing signalling molecules and altering the structure and composition of extracellular polymeric substances, potentially enhancing bacterial tolerance to environmental stressors^62^
	*Acinetobacter baumannii*	Outer membrane vesicles contribute to bacterial survival by enhancing oxidative stress tolerance^60^
	*Helicobacter pylori*	The 22-kDA outer membrane vesicle protein contributes to bacterial attachment and structural integrity, playing a crucial role in biofilm formation^61^
		OMVs play a protective role against antimicrobials (clarithromycin, levofloxacin and LL-37) and environmental stressors (hydrogen peroxide)^54^
	*Staphylococcus aureus*	Ampicillin stress induces a significant increase in MV production in methicillin resistant *S. aureus* (MRSA), with these MVs carrying ß-lactamase enzymes, enhancing tolerance and protecting both MRSA and susceptible bacteria from antibiotic killing^166^
**L-forms**	*Escherichia coli, Bacillus subtilis*	When reverted to L-forms, *E. coli* and *B. subtilis* show increased resistance to antimicrobials such as polymyxin E, meropenem, rifampicin and tetracycline^167^

In bacteria that test susceptible to an antibiotic in an MIC assay, strategic fluctuations in gene expression can allow short-term survival at the population level. Sometimes referred to as phenotypic ‘**resilience**’, this category includes **tolerance,** **persistence** and **heteroresistance**^1^. **Tolerance** is the ability of an entire bacterial population to survive transient exposure to a bactericidal antibiotic^14^, featuring slower bacterial killing compared with a fully susceptible population. Antibiotic **persistence** describes a subset of the population that can survive bactericidal antibiotic concentrations, typically represented by a biphasic kill curve. In contrast, **heteroresistance** occurs when a subset of cells within the population exhibits a higher MIC than the majority^15^, allowing it to grow despite antibiotic pressure.

**Fig. 2:**
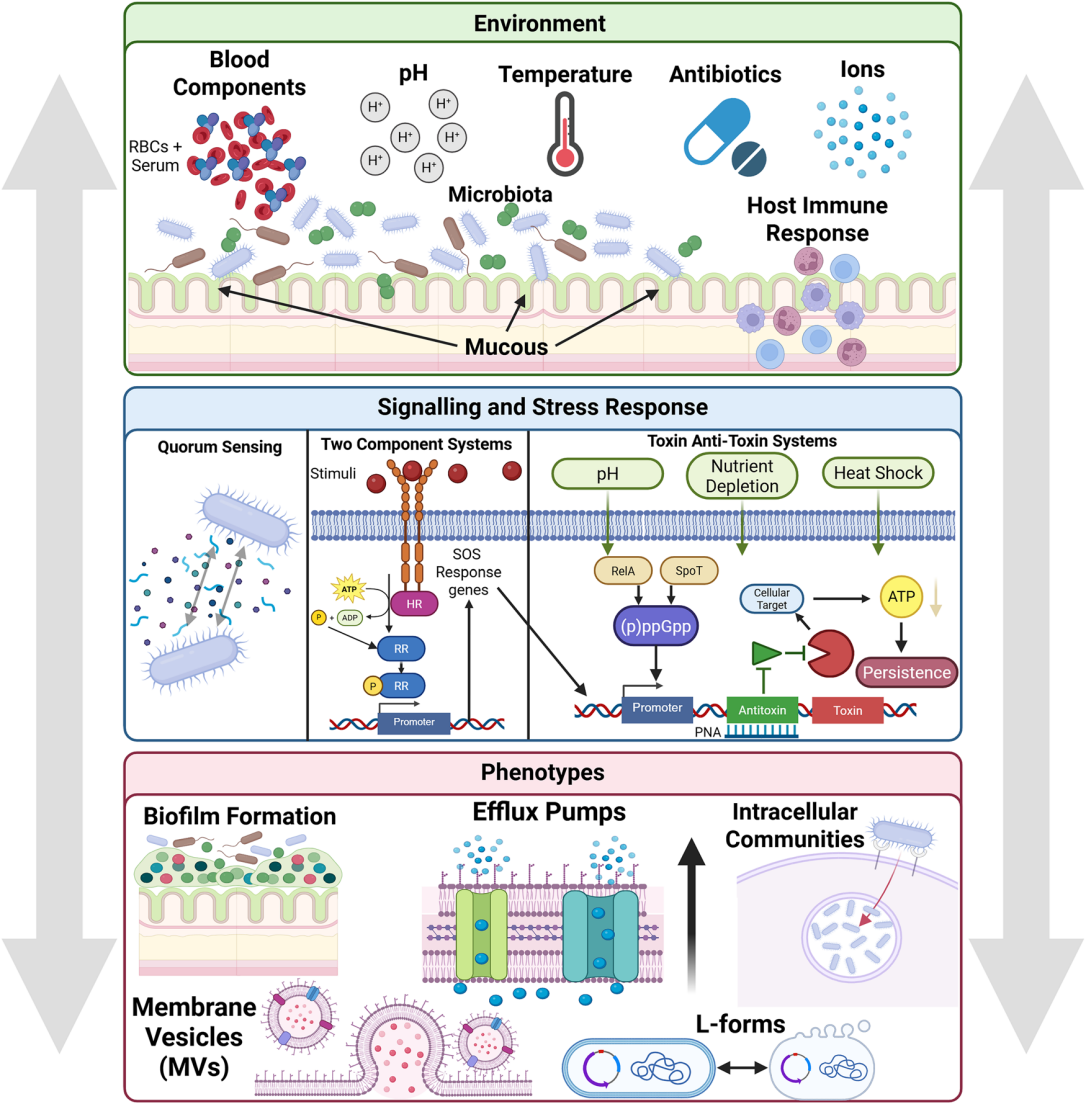
Bacteria respond to environmental cues through signalling pathways that may contribute to treatment failure through different phenotypes. Environmental cues, signalling pathways and phenotypes can influence one another and thereby be modulated in real time (grey arrows).

These temporary behaviours (Fig. 2) are regulated by a complex programme of upstream signalling systems and stress responses (blue box). These are in turn influenced by environmental signals (green box). Ultimately, the net result of these triggers may manifest in situations (pink box) that result in resilience behaviours, sometimes in tandem with more physical obstructions to antibiotics.

### Two-component systems

Bacteria rely on two-component systems (TCSs) to sense and adapt to their environment, making TCSs critical for niche adaptation and downstream gene regulation. These signalling systems are key players in AMR across diverse environmental contexts, but are less understood in the context of resilience phenotypes. TCSs can respond directly to antibiotic exposure, but environmental stressors may also induce transient changes in key global regulators, resulting in cell surface modifications, increased drug efflux, antibiotic-degrading enzyme production, and biofilm formation^16^. Typically, TCSs consist of a homodimeric histidine kinase and a cognate response regulator.

For example, in *Listeria monocytogenes*, the TCS LisRK confers selective antibiotic tolerance, particularly to ampicillin, likely by regulating penicillin-binding protein expression and modulating cell envelope properties^17^. In *Mycobacterium tuberculosis*, the TCS TcrXY, an acid-sensing system, is essential for establishing persistent infection, and silencing TcrXY attenuates persistence during chronic mouse lung infection^18^. Similarly, populations of *Escherichia coli* under nitrogen starvation conditions form a higher percentage of metabolically dormant cells through the TCS NtrBC^19^.

TCSs also facilitate major pathoadaptive phenotypes, such as biofilms (see Biofilms), which play a role in treatment resistance. The TCS LisRK mentioned above also promotes adhesion during biofilm formation. Another example is the Gac-Rsm two-component system in *Pseudomonas aeruginosa*. The Gac-Rsm cascade is a central regulatory network that mediates the phenotypic switch between acute and chronic infections^20^, controlled by the sensor kinases RetS and LadS, which modulate the activation of the GacS/GacA TCS^21^. The net result is the activation of several virulence factors involved in the lifestyle transition of *P. aeruginosa*, such as utilization of the bacterial T6SS nano-weapon, biofilm formation and motility-related adaptations^22^.

### Toxin/-Anti-toxin systems and stress response

Another proposed mechanism of bacterial persistence involves toxin/anti-toxin (TA) modules^23^. For example, *E. coli* mutants demonstrating persistence against penicillin had a gain-of-function allele, hipA7, later shown to be a toxin causing self-killing and growth arrest, relieved by the presence of its cognate anti-toxin^15^. Furthermore, overproduction *in E. coli* of the RelE toxin, an inhibitor of translation, caused a sharp increase in persister cells (defined as tolerant and often quiescent)^24^. MazF, a type II TA system in *E. coli*, was additionally shown to induce growth inhibition and persister generation^25^.

These TA modules are regulated by certain stress responses, highlighting environmental influence. These include the SOS response (facilitating DNA repair after damage), and the stringent response (which adjusts cellular behaviour during nutrient scarcity)^26^. Oxidative stress and antibiotic exposure can trigger the SOS response by upregulating genes involved in the SOS response, many of which are known to include toxin-antitoxin (TA) systems^27^. These upstream cues result in inhibition of cellular functions including transcription, translation, DNA replication and energy production, all of which result in cell dormancy. Like the SOS response, the stringent response is also modulated by certain environmental stresses including pH variation, nutrient limitation and heat shock. These triggers activate production of (p)ppGpp, mainly via SpoT and RelA, which have also been shown to activate TA modules^28^. Quorum sensing has further been linked to the regulation of TA systems via Rpos^29^.

## Downstream phenotypes

How do these regulatory networks manifest in practice? Below, we review five cases of treatment resistance where resilience mechanisms are in play, sometimes in tandem with more physical obstructions to antibiotics. Similar phenotypes to the ones described in these cases might also be achieved by other mechanisms, but here we focus on a few discrete examples.

### Biofilms

Chronic infections are often linked to microbial biofilms, which pose significant clinical challenges, whether occurring in native anatomical sites or on prosthetic material^30^. Biofilms consist of bacterial communities embedded in an extracellular polymeric substance (EPS) comprised of polysaccharides, proteins, and extracellular nucleic acids (eNAs), a structure enhancing bacterial survival and resilience to antibiotics across various infection contexts^31^. Specifically, positively charged antibiotics such as aminoglycosides may bind to, and become blocked by, negatively charged eNAs. In addition, polysaccharides can act as a permeability barrier against antibiotic penetration^32^. Thus, the biofilm structure itself can resist antibiotics even when antimicrobial tests indicate susceptibility^16^.

Bacterial populations within biofilms can also temporarily endure high doses of antibiotics by slowing metabolic processes, with some cells growing very slowly or becoming inactive (persister cells). The presence of highly metabolically active cells in the top layers and slower/inactive ones residing deeper^33^ mirrors how nutrients and oxygen are distributed and affect how different parts of the biofilm respond to antibiotic treatment^16^. Hypoxic pockets deep within the biofilm promote persister formation^16,34^. Dormant subpopulations, which are not actively dividing, complicate treatments, as reduced activity can present fewer active targets for antibiotics, which often oppose active metabolic processes, e.g. beta-lactams and cell division^35^. Persister cells can resume normal growth when treatment stops, leading to relapse or recrudescence. Important in the clinical setting, biofilms are often independent compartments with different pharmacodynamics compared with that of the surrounding tissue^36^.

Increasing evidence suggests that polymicrobial interactions and bacterial mutualism play a role in response to antibiotic tolerance and resistance. These interspecies interactions can be outlined as collective resistance, collective tolerance and exposure protection, which have been previously described^37^. In the context of this review, we argue that MICs are additionally limited because they test antimicrobial susceptibility in single species during planktonic (free-floating) growth, absent from the host microenvironment.

### Efflux pumps

Whether in planktonic populations or biofilms, efflux pumps contribute significantly to antibiotic treatment failure and multidrug resistance by regulating the intracellular environment^38^. These transmembrane proteins expel toxic substances, such as antibiotics and harmful metabolites, and are predominantly found in Gram-negative bacteria. While efflux pumps are central to classical AMR, they may also play a role in persistence and tolerance phenotypes. For example, *E. coli* persisters exposed to beta-lactam antibiotics exhibited reduced drug accumulation due to increased efflux activity, with upregulation of the tolC, acrA, and acrB multidrug efflux genes. The high expression of tolC specifically enhanced bacterial persistence^39^. TolC activity also contributed to delafloxacin persistence in *E. coli*, with induced expression of acrAB efflux genes following fluoroquinolone treatment^40^. Notably, deletion of acrB led to reduced survival rates of persister cells. Additionally, efflux pump genes in *Streptococcus pyogenes* persisters were upregulated when exposed to penicillin, along with a concurrent downregulation of genes related to protein synthesis and cell growth^41^.

### Intracellular bacterial communities

Another way bacteria may evade antibiotics is via the formation of intracellular bacterial communities (IBC), which are reservoirs that result from bacterial invasion. For example, in urinary tract infections with uropathogenic *E. coli* (UPEC)^42^, IBCs are protected from antibiotics because the tight barrier function of the urothelium limits or prevents their penetration^43^; moreover, deeper reservoirs achieve a dormant state. Metabolic flux in UPEC has been shown to regulate the quiescent state via succinyl-coA resulting in antibiotic persistence^44^. There are many other examples of difficult-to-treat bacteria with obligate or facultative intracellular lifestyles, including *Salmonella enterica*, *M. tuberculosis, L. monocytogenes* and *Chlamydia trachomatis* (reviewed by Kamaruzzaman et al.^13^).

### Membrane Vesicles

Bacteria can secrete spherical structures (20–400 nm) called membrane vesicles (MVs)^45^, which contain various cargoes and have a variety of functions^46,47^. Depending on treatment or environmental factors, MVs can be upregulated, allowing a nuanced survival response.

While MVs are known to transfer AMR genes^48–50^, they can also contribute to atypical treatment failure^45^, e.g. harbouring beta-lactamase enzymes in the case of *E. coli*^51^ and *Neisseria gonorrhoeae*^52^, or esterases associated with macrolide resistance in the case of Gram-negative bacteria (reviewed in^53^). MVs have also been implicated as decoys, decreasing the environmental antibiotic concentration and uptake of antibiotic by target bacteria^54–57^. The SOS response caused by antibiotic treatment can lead to increased MV secretion, as seen with *P. aeruginosa* responding to ciprofloxacin^58^, while different antibiotics deployed in sub-MIC concentrations can induce different quantities of MVs with different cargo^59^. Finally, MV cargo has been implicated in biofilm formation in Gram-negative bacteria such as *Acinetobacter baumannii*^60^ and *H. pylori*^61^ directly, and indirectly with *P. aeruginosa*^62^.

### L-forms

L-forms are a transient, cell wall-deficient morphotypes of normally wall-possessing bacteria which can resist wall-targeting antibiotics, increasing their tolerance and persistence. L-form formation has been shown in Gram-negative bacteria, e.g. *E. coli* isolated from recurrent UTI (rUTI) patients. L-form switching was present in response to antibiotics that target the cell wall, and may also be a mechanism of tolerance or persistence in rUTI^63^.

Environmental factors are important for L-form maintenance, as these morphotypes can only survive in osmotically stabilised conditions^64^. Reactive oxygen species (ROS) also play a role in L-form growth, with increased ROS limiting L-form formation; however, addition of ROS scavengers or anaerobic conditions contributed to L-form proliferation^65^.

## Role of the host microenvironment

The complex regulatory systems described in Mechanisms of treatment resistance section, and their practical outputs in Downstream phenotypes section, are broadly influenced by the larger environment (Fig. 2, green box). While the broader milieu can affect bacterial phenotype and contribute to classical AMR by providing selective pressures, less well understood is how the environment can affect treatment response in other ways. Fluctuations in host anatomy, physiology, metabolic function and cellular components can lead to homoeostatic imbalance, which in turn can affect drug pharmacokinetics/pharmacodynamics (PK/PD)^4,66,67^. Such fluctuations include blood composition, pH, microbiota and mucous composition, which are elements not usually reflected in in vitro diagnostic testing^4^.

### Blood composition

Systemically administered antibiotics enter the bloodstream either directly (e.g. through the intravenous route) or indirectly following absorption (e.g. through the gastrointestinal tract), where they circulate until they are bound to blood cells or plasma proteins, which can prevent effective drug function^68^. As protein binding to drug increases, the overall pharmacological efficacy decreases as the amount of active drug left in circulation is lowered^4,67^. Another factor is that erythrocytes contain iron, which promotes bacterial growth. Indeed, the addition of lysed erythrocytes into cultures of *E. coli*, *Staphylococcusaureus* and *P. aeruginosa* raised the MIC of meropenem, ciprofloxacin and tigecycline^68^; moreover, significant differences in MIC for oxacillin, ampicillin and moxifloxacin occurred in *S. aureus* when cultured with albumin^68^. In addition, the inclusion of albumin to bacterial cultures led to a significant decrease in bacterial killing by moxifloxacin^68^. Similarly, *E. coli* and *Streptococcus pneumoniae* treated with serum albumin showed 8-fold higher MIC values for ceftriaxone^69^. Human serum has also been shown to trigger antibiotic tolerance in *S. aureus*^70^.

### pH

Maintenance of an acid-base balance is a major component of homoeostasis, and variations of H^+^ concentration can alter protein structure and function, causing a difference in drug bioavailability^4^. Acidic environments occur naturally (e.g. in the vagina) as a defence mechanism to prevent bacterial colonisation. In addition, acid stress responses are essential for survival^71,72^. During infection, local pH can change due to the presence of bacteria, with concomitant alterations in cell metabolism affecting antibiotic function, which could affect treatment outcomes^73^.

It has been noted that fluctuating pH is not reflected in MIC tests, which could affect their results^74^. Indeed, urinary pH is known to affect antibiotic efficacy^75^. For example, in one study, patient-relevant acidification in MIC tests increased the susceptibility of 71% of the bacterial isolates^76^. In the case of ciprofloxacin, the impact of acidic pH on *E. coli* susceptibility was due to the positive and negative charge on the drug and its zwitterionic nature^76^. Another study focusing on UPEC resistance to trimethoprim sulfamethoxazole also reported that fluctuating urine pH affected the MIC^77^.

### Other environmental factors

Aside from its pH, the composition of urine, including salt and urea, may affect the transcriptome of urinary pathogens^16^ as well as their response to antibiotics^78^. The gut microbiota is known to affect drugs generally, with most research focusing chemotherapeutics^78^; while not well-described, antibiotics may also be processed and altered by commensal microorganisms. Media mimicking mucus has been used to show how this milieu affects *P. aeruginosa* during cystic fibrosis infection^79^, while others have studied the effect of glucose on growth and co-culture of *S. aureus* and *P. aeruginosa* in artificial sputum medium^80^.

## Beyond the MIC: Challenges and advances in more physiological antimicrobial susceptibility testing platforms

In addition to the gold-standard MIC, other methods are also employed in clinical settings, relying either on phenotypic outputs (e.g. growth, death), or on omics-based identification of resistance (Table 1). More recently, faster methods have been developed that detect more advanced parameters, although they are expensive. For example, the Accelerate Pheno System identifies bacteria by gel electrofiltration and fluorescence in situ hybridisation, measuring bacterial growth using microscopy to infer MIC values^81^. Resistell technology uses atomic force microscopy (AFM) to measure bacterial nanomotion, a proxy for viability^1^. Finally, the Sysmex PA-100 System deploys nanofluidics and imaging to determine cell growth. Yet none of these recapitulates a more physiological microenvironment^82^.

### Media mimicking

The most basic improvement that could be made in planktonic antimicrobial sensitivity testing would involve supplementing, or fully replacing, the standard bacterial growth medium used for MIC (e.g. Mueller-Hinton broth) with the relevant bodily fluids or their synthetic equivalent^83,84^. Heithoff et al. showed that ~15% of MIC tested in more physiological media predicted a shift across the clinical breakpoint, altering susceptibility classification^84^. Similarly, by using sputum-mimicking media, a reduction in antimicrobial susceptibility of *Mycobacterium abscessus* was observed in comparison with standard medium^85^. As previously mentioned, the host microenvironment might trigger phenotypic adaptations that lead to bacterial tolerance or increased antimicrobial susceptibility. For example, the presence of serum in growth medium triggered tolerance of *S. aureus* against daptomycin by two distinct mechanisms, leading to peptidoglycan accumulation and increased cardiolipin concentration in the bacterial membrane^70^. Transcriptomics analysis of *P. aeruginosa* isolated directly from a human infection was compared with laboratory growth conditions to explore this issue. Key genes related to antimicrobial resistance were found to be upregulated in the human host infection in comparison with the in vitro settings, which potentially could explain why AST assays often underestimate resistance in the clinical settings^86^.

As a potential case in point, in a side-by-side comparison, the Mast Uri® System, where patient urine is inoculated directly onto antibiotic-containing agar, was found to call resistance more frequently than did standard MIC^87^. Although the authors interpreted this finding as the Mast Uri over-estimating resistance, it is equally possible that the MIC was under-estimating it. The different results may stem from the fact that, unlike a standard MIC, where a colony is selected after overnight growth and adaptation to agar-containing standard culture medium, the inoculated bacteria come straight from the patient, where their metabolism is primed for the nutrient-poor urine environment.

While enriched medium can recapitulate some physiological aspects of planktonic conditions, the reality is that many infections in a clinical setting involve biofilms^88^, and of course, host cells, alongside other external factors such as fluid flow^89^. There is therefore great interest in developing more physiological platforms to test antibiotics in these more complicated microenvironments.

### Biofilm susceptibility testing methods

Typically, acute infections are caused by planktonically growing bacteria, while biofilm-associated bacteria are involved in chronic infections. Indeed, in humans, 80% of chronic infections are thought to be caused by biofilm-associated bacteria^90,91^. Due to their unique features, biofilm-associated infections manifest between 100-1000-fold increased antimicrobial resistance compared with planktonically growing bacteria^88,92^.

**Fig. 3:**
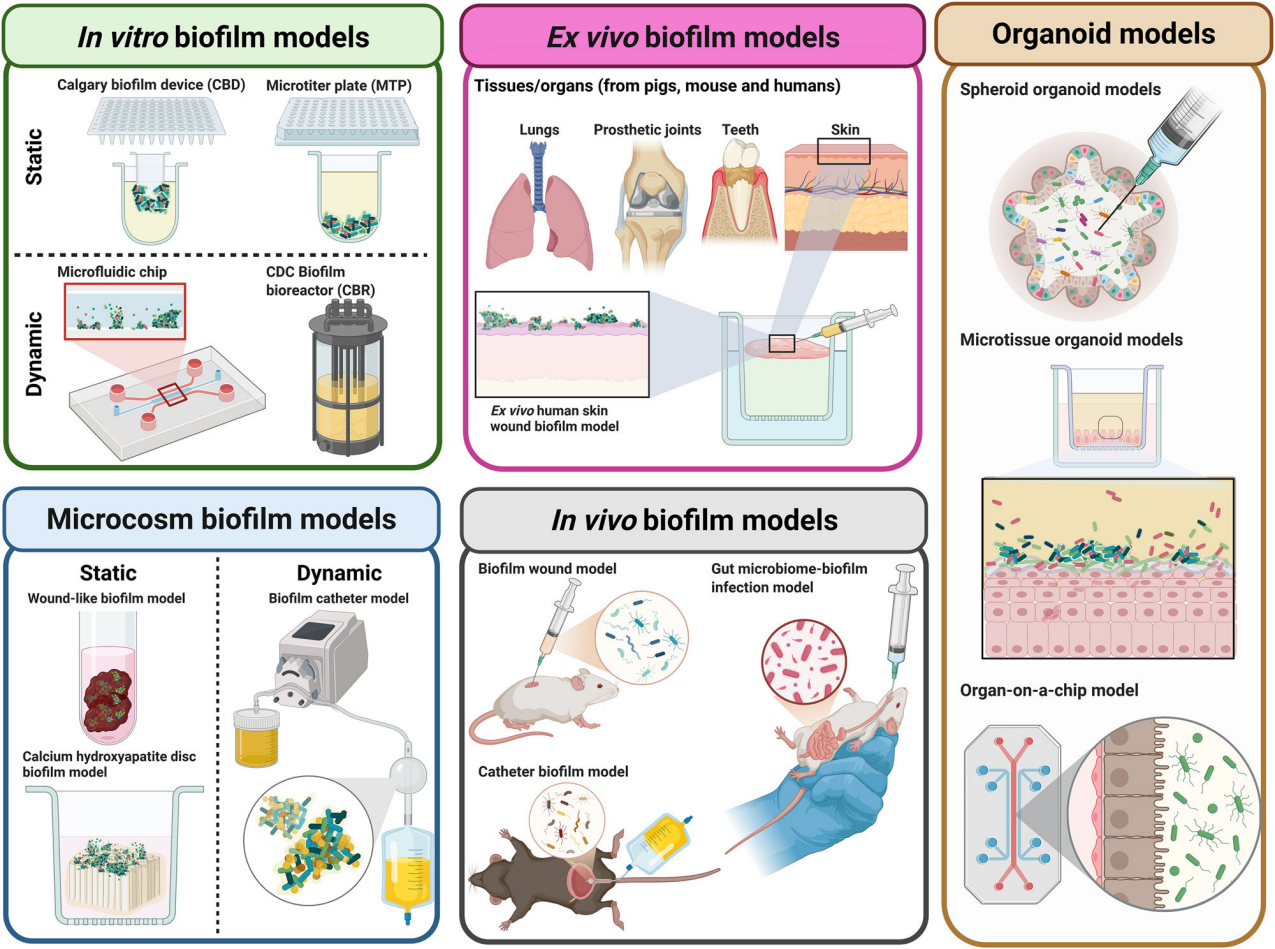
More complex models, incorporating biofilms and/or flow, mammalian cells, excised tissue or animals. These model have been subcategorised depending on the complexity of the platform used to grow the bacterial biofilm (in vitro, microcosm, ex vivo, organoid and in vivo model).

Different types of biofilm models are summarized in Table 3 with their pros and cons, alongside examples, and illustrated in Fig. 3. Generally, these methods involve growing biofilms on different abiotic substrates, such as agar, plastic, glass or beads, which can be either static or embedded in a dynamic flow system, such as a bioreactor or microfluidic chip^93,94^. A significant challenge in biofilm susceptibility testing methods is standardization, as well as determining whether a given methodology yields clinically relevant results^95^. Neither the European Committee on Antimicrobial Susceptibility Testing (EUCAST) nor the Clinical and Laboratory Standards Institute (CLSI) have established standardized definitions of biofilm endpoint parameters/MIC^96^. In the biofilm field, different susceptibility endpoint parameters have been reported (Table 4). However, some of the terms are often used interchangeably in the literature, causing confusion^16,96,97^.

**Table 3:** Relatively simple biofilm models

Model	Advantages	Disadvantages	Examples
**In vitro biofilm models**			
**Static**	• Cost-effective, reproducible, rapid and easy • High-throughput nature useful for investigating many parameters or many strains at once • Have allowed meaningful research findings (e.g. biochemical signals regulating switch between planktonic and biofilm growth) • Can investigate biofilm biomass and antimicrobial susceptibility testing	• Little detailed insight into biofilm architecture or cellular viability within the biofilm, • Low control over critical environmental variables like flow rate, shear stress, and nutrient gradients • Relies on biofilm adherence to plastic/glass surface, which may not mimic other substrates like prosthetic joints, teeth, wounds, epithelia etc., limiting applicability • Nutrient and oxygen availability limited to initial conditions	• Colony biofilm method on agar^168^, used to study early stages of biofilm formation and antibiotic resistance • The microtiter plate (MTP)^169^ allows quantification of biofilm biomass, but can be confounded by sedimented cells on bottom of wells. • Calgary biofilm device (CBD)^169^: quantifies biofilm formed on lid pegs • Biofilm Ring Test (BRT)^169^, used to study early-stage biofilm formation, in which a magnetic field is applied to determine the immobilisation of magnetic beads due to biofilm
**Dynamic**	• Biofilms maintained in laboratory settings for weeks, unlike static models • Used to investigate chronic or mature biofilms • Used to investigate dynamic changes of biofilms subjected to shear stress • Generally, biofilms are grown on removable coupons (CBR model) or microscopy slides (DFBR model), allowing biofilm architecture investigation with different types of microscopy. • Microfluidic technologies enable real-time analysis of dynamic processes with precise control over hydrodynamic parameters compared with conventional flow bioreactors on the macroscale^170^ • Microfluidic devices allow investigation of structural dynamic changes of biofilms subjected to shear stress	• Protocols usually are not standardized • Relies on specialised and expensive equipment	• CDC Biofilm reactor (CBR), Drip Flow Reactor (DFBR) and Rotating Disk reactor (RDR) are standardized by the American Society for Testing and Materials (ASTM), used to investigate the antimicrobial efficacy of drugs against *P. aeruginosa* or *S. aureus* biofilms in hydrodynamic conditions, extensively reviewed elsewhere^171^ • A microfluidic device showed that shear stress triggered initial adhesion by enhancing nutrient availability and promoted dispersal opportunities but inhibited biofilm development during the maturation and dispersion phases^172^ • BiofilmChip is a screening platform to test antibiofilm drugs using a microfluidic platform with an integrated interdigitated sensor. Biofilms can be assessed directly using confocal microscopy and electrical impedance spectroscopy on same platform^173^

**Table 4:** Biofilm assay endpoint terms

Term	Definition
The minimal biofilm eradication concentration (**MBEC**)	The lowest concentration of a compound that prevents visible growth, including eradication of any persister cells
The biofilm bactericidal concentration **(BBC**)	The lowest concentration of an antibiotic that causes a 99.9% reduction of a biofilm culture (a reduction of 3 log_10_ in CFUs compared with untreated biofilm controls)
The minimal biofilm inhibitory concentration (**MBIC**)	The minimum concentration of a drug that prevents any time-dependent growth in the average number of viable cells within a biofilm, normally measured by recording the OD_650_ difference of ≤10% after 6 h of incubation
The biofilm prevention concentration (**BPC**)	The minimum concentration of a drug at which the cell density of a planktonic culture is sufficiently lowered to inhibit biofilm formation

The main limitation of biofilm testing methods is the uncertain validity of the model used^94^. Although biofilms have been studied extensively in vitro, they may not accurately mimic biofilms found in vivo in chronic infections^93^. The lack of chemical/physical host-microenvironment, alongside the relatively short infection set-up of in vitro experiments, are additional key differences^98^.

### More complex infection models

In addition to studying biofilms in isolation, the next logical step is to include more physiological components. Table 5 and Fig. 3 summarise these types of models^93,94,99,100^, all of which can also foster biofilms. One such model is the microcosm model, which includes the nutrients and structures present in a normal infection. As an example, a saliva-derived polymicrobial biofilm model on a titanium surface was developed to test antimicrobial therapies; even when the treatments were efficacious, the polymicrobial communities re-established after two days with an altered microbial diversity^101^. Secondly, ex vivo models deploy tissue excised from an animal or human. For example, porcine skin was used to study *S. aureus* wound infections, and porcine lung, to study *P. aeruginosa* in cystic fibrosis; in both cases, the bacteria exhibited a higher antimicrobial susceptibility compared with their respective MICs, even when performed in mimicking host media^102^. Thirdly, organoid/microtissue models involve biomimetic microtissue or organoids which are grown and then infected experimentally. For instance, we developed the 3D human urine-tolerant urothelial model (3D-UHU), which allows infections in the presence of 100% urine^103,104^; this supports the formation of intracellular reservoirs as well as biofilms on the urothelial surface, and can be used to test antibiotic response. Sharma et al. reported a dynamic urothelial on-chip model incorporating flow and stretch parameters in which intracellular bacteria were more resistant to antibiotics^105^. Finally, in vivo models, where infected animals are studied, are arguably the most complex, and have been heavily relied upon, but for some parameters, such as direct host cell/pathogen interactions, their relevance may not be greater than human ex vivo or organoid/microtissue models^94,106^.

**Table 5:** More complex infection models

Advantages	Disadvantages	Examples
***Microcosm*** **biofilm models**		
• Models can be static or dynamic systems • Include additional features, such as cells, materials, or nutrients to better mimic the pathological environment of biofilm infection in human body • Recapitulate natural biological structures and host microenvironment where biofilms typically form	• Protocols not standardized • Lack of validation with clinical data • Tend to be low high-throughput and more expensive in comparison with simpler in vitro models (Table 3)	• Artificial sputum medium (ASM)^174^ mimics complex microenvironment involved in cystic fibrosis (CF) lungs. It contains nutrients such as sugars, amino acids and irons, supplemented to approximate in vivo concentrations in human sputum samples, as well as DNA, N-acetyl glucosamine and mucin to modulate the physiological conditions that allow biofilm aggregates to form with a similar architecture as those observed in CF patients^175^ • The Lubbock Chronic Wound Biofilm (LCWB) model mimics biological and environmental conditions of chronic wounds by incorporating elements of chronic wound physiology, specifically chopped-meat-based medium and plasma/serum. Model supports polymicrobial communities including pathogens such as *P. aeruginosa* and *S. aureus*, traditionally difficult to co-culture in vitro. Supports growth of anaerobes, which are crucial constituents of such infections^176^ • Collagen-based in vitro model with synthetic wound fluid (SWF) and a 3D-collagen matrix mimics soft tissue infection, and showed important hallmarks such as bacterial embedment and antibiotic tolerance^177^ • Tissue-engineered skin equivalent (Graftskin) used to investigate biofilm formation as an alternative to ex vivo skin models^178^ • Dental biofilm model with high-throughput microfluidics using human saliva to grow multi-species oral biofilms closely mimicking in vivo conditions to assess drug efficacy^179^. Can study multi-species biofilms using same device^180^ • MTP (microtitre plate) device acts as physical substrate to support growth of biofilms on various materials, such as medical-grade silicone titanium, to mimic prosthetic joint infections^169^ • In vitro osteoblast human cells culture supernatant showed increased biofilm formation of *S. aureus*, emphasising influence of the host microenvironment in biofilm formation and, indirectly, antimicrobial response^181^ • Artificial urine medium (AUM)^182^, synthetic human urine (SHU)^183^ and multi-purpose artificial urine (MP-AU)^184^ have been developed to increase physiological relevance of UTI models • In vitro catheter-associated UTI (CAUTI) biofilm model using AUM and indwelling catheters was used to test efficacy of antibiotics and phage cocktails against *K. pneumoniae* biofilms^185^ • FlexiPeg biofilm device, a 3D-printed design of classic Calgary device, allows modularity (individual peg lids-removable), reusability (autoclavable materials) and the selection of desired coating materials (for example, silicon to mimic the materials from catheters employed in the clinic)^186^
**Ex vivo biofilm models**		
• Provide more accurate representation compared with purely in vitro systems • Recapitulate natural biological structures and host microenvironments • Some tissues remain immunocompetent, a key aspect missing in the aforementioned models	• Protocols not standardized • Lack of validation with clinical data • Tend to be low-throughput and more expensive in comparison with in vitro models	• Ex vivo skin models using porcine skin or left-over human skin obtained from surgical interventions are used to mimic chronic wounds and burn infections^187–189^, with immunocompentency (resident immune cells) mimicking host infection response • Porcine model of cystic fibrosis lung infection recapitulated some key aspects of *P. aeruginosa* infection, metabolism and biofilm architecture, closer to sputum samples obtained in the clinic^114^ • Dental biofilm model using human enamel blocks infected with biofilms obtained from human saliva are used to investigate root caries and the ability of *C. albicans* biofilms to cause demineralisation^190^
**3D-microtissue/organoid models**		
• Advances in cell biology and bioengineering have allowed the development of 3D-microtissue models using human stem or stem-like cells that mimic the function and structure of organs • The FDA approved such models as valid pre-clinical data to circumvent animal work in some cases^191^ • These tissue-mimetic models are useful platforms for modelling the host microenvironment, closely recapitulating key aspects of human infections^192,193^	• Protocols not standardized • Lack of validation with clinical data • Tend to be low high-throughput and more expensive in comparison with simpler models	• Human lung organoid model derived from human biopsies are used to investigate cell invasion of *P. aeruginosa* at molecular level and characterise biofilm formation within the human lung epithelium^194^. Another lung organoid model showed that biofilm formation increased antibiotic tolerance at the mucosal surface^195^. • A 3D urine-tolerant human urothelium (3D-UHU) microtissue model was developed to investigate various aspects of UTI host/pathogen interactions in the bladder^103^, in an interface that includes both the host urothelium and 100% human urine; *P. aeruginosa* and *Proteus mirabilis* biofilms formed readily, with a crystalline aspect reminiscent of clinical samples^104^. As an example of a dynamic system, another urothelial chip model featured flow and stretch dynamics to mimic bladder filling and voiding^105^ • Gut organoids to investigate bacterial-epithelial interactions have been reviewed extensively elsewhere^196^. As one example, a model containing a gut epithelial layer and a myofibroblast layer separated by a polycarbonate layer or 3D scaffold was used to investigate *Clostridium difficile* pathogenesis in anaerobic conditions^197^. Furthermore, a gut-chip model has been used to investigate biofilm formation of *Lacticaseibacillus rhamnosus* and host-mucus production response^198^. • Vaginal organoids have been developed using 3D cervical epithelial cells to investigate bacterial vaginosis, and infected with polymicrobial communities to investigate the effect of health-associated commensals such as *Lactobacillus crispatus*^199^.
**In vivo biofilm models**		
• These models clearly provide the highest degree of complexity for studying biofilms	• The expense, infrastructure, ethical considerations and governmental regulation of higher animal models present a high bar for uptake^94^. • Host used might not fully recapitulate the host-microenvironment in humans (reviewed recently in the case of UTI models^106^).	• In vivo mouse wound model against *P. aeruginosa* biofilms demonstrated antibiofilm properties of meropenem in combination with enzymes that degrade biofilm matrix^200^ • Primates, pig, rats and especially mice have been extensively used to investigate UTIs; key pathogenic strategies have been identified and therapies to treat UTIs have been explored^201^; additionally, mouse models to investigate catheter associated UTI (CAUTI) have been used^202^

However, recapitulating more physiological infections, including model development, validation and real-world execution, are challenging^94^. To date, most biofilm therapy testing is still performed using simple in vitro biofilm models, or animal models that do not fully recapitulate the human host-microenvironment^100^. The discrepancies between antibiofilm drug efficacy and clinical trial performance strongly suggest that microenvironment matters in therapeutic development as well as in susceptibility testing^95^.

In choosing which system might be most appropriate, a further complication is that some bacterial infections, particularly those resulting in sepsis, involve multiple niches, with bacteria present in the bloodstream as well as in the originally infected site^107^. It is therefore a challenge to know which physiological testing platform might be the most appropriate. It must also be acknowledged that increased complexity will likely be more time- and resource-intensive, which may not be possible in some diagnostic settings.

## Clinical relevance and conclusions

If a testing platform is not sufficiently nuanced to recapitulate the correct infection microenvironment, it seems logical that it might struggle to accurately predict clinical outcome. But what is the evidence for this? Taking a big-picture perspective, treatment failure rates can serve as a proxy that diagnostic techniques are not always optimal. For example, recurrence rates for urinary tract infection are worryingly high^108^. While uncomplicated UTI tends to be treated empirically and not all samples undergo AST, not all treatment failure is resistance-mediated.

In addition, it has been shown that the predictive value of AST methods on treatment success is relatively low on immunocompromised patients, patients treated with multiple antimicrobials, and patients with infections caused by polymicrobial communities^109^ (which demonstrate synergy in resistance^110,111^). A systematic review revealed lack of evidence of AST methods as a predictor of clinical outcomes in cystic fibrosis antimicrobial treatment^112^.

Given the documented discordance between AST and outcome, it would therefore be of great interest to examine studies that compared the predictive value of traditional testing to more advanced methods; unfortunately, such studies are vanishingly rare and more research is needed. As one example, a randomised controlled clinical trial to compare the utility of biofilm antimicrobial susceptibility testing versus MIC in treating pulmonary exacerbations in cystic fibrosis (CF) patients revealed no significant differences in pulmonary bacterial loads nor clinical outcomes^4^. The antimicrobial biofilm test was a Calgary device where biofilms of *P. aeruginosa* were grown in a rich media. Although this methodology offers high-throughput screening in clinical microbiology settings, it grossly oversimplifies the CF environment. Lung biopsies of people with CF reveal biofilms suspended in bronchial mucus, a sponge-like mass filled with mucus, alginate and/or lung fluid. These are substantially different to biofilms observed in vitro^113^. Hence, it is likely that in vitro biofilms do not accurately mimic CF biofilms and the antibiofilm susceptibility test performed offered no substantial advantages in comparison with traditional antimicrobial testing^114,115^. However, this example is valuable firstly in that it shows again that traditional testing cannot always predict treatment outcomes, and secondly that one cannot assume that only incremental complexity in modelling will make a difference.

In summary, recapitulating the host microenvironment to allow for more meaningful host-pathogen-drug interactions are key to advancing fundamental knowledge about the biology of treatment failure, and to increasing the pipeline for badly needed new alternatives to antibiotics. In parallel, the future of antimicrobial susceptibility testing likely lies in increasing the complexity and physiological relevance of platforms, while balancing these advances with the need to achieve high-throughput, point-of-care (or near point-of-care) timelines and manageable healthcare expense. We acknowledge that this balance will not be trivial to achieve. Finally, we need clinical studies and robust data to back up the ultimate clinical usefulness of newly developed tests.

## Data Availability

No datasets were generated or analysed during the current study.
